# A Lesson from Ovariotomies

**Published:** 1879-06

**Authors:** J. T. Everett

**Affiliations:** Sterling, Ill.


ARTICLE II.

A LESSON FROM OVARIOTOMIES. By J. T. Everett, A.M., M.D., Sterling, Ill.

The knowledge acquired by failures is food less palatable than nutritious. It is more unpleasant to publish our unsuccessful than our successful operations. Where, however, a lesson is to be learned, they are just as much the property of the profession as our most brilliant achievements.

*Case I*, was performed November 17, 1872, and reported in the *Chicago Medical Examiner* for April, 1873. Patient, 41 years of age, complaining of pain in the back, increased nervousness and epigastric neuralgia; lump in left ovarian region the size of an adult head. Bimanual palpation gave distinct fluctuation and wave motion, and the hypodermic needle withdrew clear, straw-colored serum, containing albumen and ovarian cells.

On the 16th, an active cathartic was given, and after the bowels had been thoroughly evacuated, a suppository of 0.6 gms. pulv. opii was inserted. The bladder was then entirely emptied by means of the catheter, and with the assistance of Drs. Hunt and Newton, I proceeded to operate in the following manner:

The patient having been fully anæsthetized, an incision eight centimeters in length was made along the linea alba, midway between the symphysis and the umbilicus. On reaching the peritoneum, this membrane was raised and nicked, and divided upon the grooved director. The sac was next examined and found to be a monocyst, and non-adherent. The sac was next evacuated, fortunately without the escape of a drop of the cystic contents into the peritoneal cavity. The tumor was then lifted out of its bed, the pedicle transfixed by a needle carrying a double carbolized animal ligature, which was tied each way and cut short. The pedicle was then pared off and dropped into the abdominal cavity. The previous superficial hæmorrhage from the lesser branches of the epigastric artery, with their accompanying veins, having been entirely controlled at the time of their division, there was no perceptible exudation of any kind into the peritoneal cavity. This was next carefully sponged out with warm carbolated water, to make assurance doubly sure, and to guard against any possible septic contamination. The abdominal walls were next brought together and secured with quilled sutures of silver wire, a film of carbolated collodium placed over the incision, this in turn covered with a layer of cotton saturated with carbolized oil, and over all a wide flannel bandage. The patient was kept under the influence of morphia administered hypodermically for several days, with a free use of tr. ferri chloridi, and quinia.

On the 23d, the bowels were evacuated. On the 24th, a slight puffiness appeared at the lower end of the incision, and on removing the dressing about 128 CC. of clear serum escaped. The orifice was again closed, and healed rapidly.

Recovery fully established at the end of the fourth week. The sac and contents weighed nearly 5 kilograms.

*Case II.* Was called September 3, 1872, to see Mrs. A. M., aged 32 years, multipara, anæmic, with marked specific symptoms. Complained of severe epigastric pains, neuralgia and bearing down in back and bowels. Examination revealed an eroded os, from which oozed a thick ropy secretion. Distended abdomen, of the size of pregnancy at the 7th month. Circumference at hips, 91 Cm.; circumference at umbilicus, 108 Cm.; circumference at ensiform cartilage, 81 Cm. Bimanual palpation gave distinct fluctuation and wave motion.

I inserted three insulated silver needles 14 Cm. into the most prominent portion of the cyst and attached the negative pole of a 20-cell zinc carbon battery, the positive pole being placed over the sacro-lumbar junction. This current was allowed to flow through the tumor for 5 minutes.

September 8.—Patient has felt no bad results from the treatment, but owing perhaps to the constitutional and local treatment is feeling stronger and has less pain. On measurement, the umbilical diameter was found to have decreased to 81 Cm. Same treatment.

September 15.—Found patient still better; umbilical diameter 71 Cm. Patient gradually improved until entire recovery took place. Was lost sight of for three years, when she was again examined, and no trace of cyst was discoverable except an enlarged ovary, with some bloating and pelvic adhesions and tenderness.

*Case III.* Saw Mrs F. H. May 10, 1873; multipara, aged 26; general health good, but suffering much mental trouble on account of a growth in left ovarian region, the size of the adult head. Examination revealed womb nearly normal; bi-manual palpation gave clear fluctuation and wave motion.

To confirm diagnosis of ovarian cyst, the hypodermic needle was thrust into the most prominent part of the tumor and withdrawn with clear fluid about the consistence of glycerine, which showed, under the microscope, well-defined ovarian corpuscles, and gave the reaction of albumen, and traces of paralbumen.

At first sitting, a needle was introduced 10 Cm. into the sac, and a 20-cell current passed for five minutes, as in the preceding case. 16th.—Same treated repeated. 22d. Same treatment. 29th. Tumor less in size; repeated treatment.

June 5th and 12th.—Treatments repeated; tumor reduced one-half in size, and more dense to the touch; catamenia appeared on the 15th, more profuse than usual. 20th.— Repeated treatment; tumor still decreasing slowly. Eight more treatments were given at weekly intervals before the growth entirely disappeared. Patient has since borne a child, and is now enjoying good health.

The query arises, why should this small tumor require fifteen sittings for its destruction, while one nearly twice the size disappeared rapidly after only three treatments? Was the previous case one of monocystic tumor, and were the whole contents changed in character by the electric current, and thus more rapidly absorbed? And in the present case, was each separate cyst of a polycystic tumor decomposed and absorbed before the next could be reached and cured?

*Case IV.* (*a*) December 8th, 1873, saw Mrs. S. W. Multipara, formerly in robust health; but now presenting the peculiar facial characteristics of ovarian trouble. Had noticed the growth for four years, with constantly increasing symptoms of pain in the back and loins, with epigastric neuralgia, great nervousness and exhaustion. Vaginal examination revealed soft, flabby os; uterus pushed over to the right side and low down. Tumor extending from true pelvis to ensiform cartilage. Umbilical circumference, 124 Cm. Inserted three platinum needles 18 Cm. into the cyst and passed a 30 cell current for 20 minutes, in the same manner as in the two previous cases. The patient was now allowed an hour's rest, and as I had to return to the city, the current was again used in like manner for 30 minutes. Patient was left, after ordering a supporting treatment, and rapidly improved. At the end of the third week the umbilical diameter reached 76 Cm. Tumor rapidly disappeared, and at the end of the first year she considered herself perfectly well.

In the spring of 1878, five years from the time of the supposed cure, I reported the last three cases to Dr. Paul F. Mundé, who incorporated them in his list of cases successfully treated by electrolysis, in the paper which he prepared on "Electrolysis of Ovarian Tumors," for Vol. II of the Transactions of the American Gynæcological Society.

The following case shows that the cure was not permanent:

*Case IV* (*b*).—Was called Sept. 30th, 1878, to see Mrs. S. W., whom I had cured (?) in 1873 by electrolysis. Found that the tumor had returned with all the concomitant symptoms of true ovarian tumor, and was of the size of pregnancy at term. Hypodermic needle withdrew a clear, viscid, straw-colored fluid. As the patient lived 20 miles from the city, and as circumstances precluded my making another visit — although armed only with an ordinary pocket case of instruments — I concluded to operate. With the assistance of Drs. Freer, Smith and Fergus, I operated in the usual manner.

The bowels having been thoroughly evacuated by a copious injection (there being no time for a cathartic to act), a hypodermic injection of .02 Gms. of morphia was given, and the patient thoroughly anæsthetized by Dr. Freer.

The urine remaining in the bladder was drawn off and an incision 10 Cm. in length was made along the linea alba, midway between the symphysis pubis and the umbilicus. Upon reaching the peritoneum, this membrane was divided upon the director, after having controlled the superficial hæmorrhage from the twigs of the epigastric vessels.

The sac was now seized with two pairs of rat-toothed forceps, one on each side, and held well up against the cut margin of the peritoneum, and then divided with the bistoury. Fortunately but very little of the cyst contents entered the abdominal cavity. After the cyst was nearly emptied, it was drawn out, the pedicle transfixed with a needle carrying a double silver wire ligature, which was cut and tied each way. The pedicle was cut short and dropped back ; the abdominal cavity carefully sponged out with warm carbolated water, and the walls approximated with silver wire interrupted sutures. Over all a coating of carbolized oil, on cotton, and this retained in place by a flannel bandage. The sac and contents weighed 13.6 kilograms.

The patient was placed in bed, and left in the care of Dr. Freer, who has just furnished me with the subsequent history of the case.

Patient kept well under the influence of morphia for the first two or three days, with free use of iron and quinine. On the sixth day, bowels moved spontaneously; sutures removed on the 10th, leaving good, strong cicatrix. Patient did not have a single untoward symptom through the whole time, but made a rapid recovery, and is now apparently as well as ever, with slight tenderness over the line of the incision. Menstruation appeared at the second month, and has recurred regularly ever since.

*Case V.*—Was called, Dec. 11th, 1878, to see Mrs. A. C., aged 52, multipara, naturally very robust. I learned that patient had been troubled somewhat with pelvic pains for several years.

About three years ago she first noticed abdominal enlargement, which at first was ascribed to natural causes ; but after reaching full period and no change taking place except a constant increase in size, some uneasiness was felt, but still no advice was sought until about two months previous to my visit, when symptoms of a low type of fever setting in, a physician was summoned, who prescribed symptomatically, and treated the case very ably, but still failed to arrive at a full diagnosis of the case. The patient grew gradually worse, notwithstanding the well directed efforts of the physician to build up the patient, until, at my first visit, the distension of the abdomen was such as to interfere materially with respiration and nutrition. The patient had not been able to assume the recumbent position for the last three days. Had not slept any for 48 hours. Exhaustion and carbonization of the blood had greatly impaired the intellection. In fact the appearances were, that the patient could not last many hours without immediate relief. On making out the diagnosis, which was not difficult, I informed the family of the same, and advised aspiration as a palliative measure. This was at once consented to, and I immediately drew off 6.13 kilograms of clear, transparent, ropy fluid. This sac being emptied, and the needle withdrawn, the patient lay back on the bed, gave a sigh of relief, and said, "Now I feel easy, and can sleep." She at once dropped into a quiet sleep, and rested nicely until morning. The next morning I aspirated another cyst, but the contents were too viscid to pass through the needle; therefore I withdrew it, and in its place inserted a trochar having the capacity of .03 Cm.; through this escaped only a small quantity, when it became clogged by a mass of floating fibrin, and the attempt was given up and the trochar withdrawn.

I now left the patient, with directions for tonic and stimulating treatment.

Dec. 17th I saw the patient again, and notwithstanding the gravity of the case, found her somewhat improved. Again aspirated with large needle and withdrew 6.59 kilograms of thick, ropy contents, of the consistence and appearance of soft soap. The friends requested an early operation, they having been previously made acquainted with the probabilities in the case.

Dec. 21st, I found patient much the same, and evacuated two more sacs of 2 kilograms each. I informed the family that, in the present condition of the patient, I could not advise an operation, but as that held out the only chance of saving or prolonging the patient's life, I would operate if they requested. This being their wish, I visited the patient Dec. 24th, finding her in the same condition as on the previous occasion. With the assistance of Drs. Gillespie and Mosher, I proceeded to operate in the following manner:—

The bowels and bladder having been previously evacuated, a suppository of opium was introduced. The patient was then anæsthetized with chloroform by Dr. Gillespie. The spray apparatus was consigned to the care of Dr. Mosher, who also rendered such operative assistance as the case required. On opening the abdominal cavity, the adhesions between the peritoneum and the sac were so dense and extensive that it required the utmost care to distinguish the one from the other.

The adhesions were at length carefully broken up with the hand, and, on attempting to thrust the "Spencer Wells trochar" into the most prominent sac, its friable walls ruptured like so much wet paper, and allowed the contents to escape into the pelvic and abdominal cavities. The remaining sacs, four in number, were then rapidly evacuated, and the tumor lifted out; its pedicle was transfixed by a needle carrying a double silk ligature, which was cut and tied each way.

The pedicle was then cut off 1 Cm. from the ligature, and dropped into the abdominal cavity, the ends of the ligature being brought out at the lower angle of the wound.

The walls were carefully sponged out with warm, carbolated water; and, after having been exposed to a strong light, to make sure that all hæmorrhage from the ruptured vessels in the adhesions had been entirely controlled, were brought together with nine interrupted silver sutures. Between each a long, adhesive strap enclosed half the abdominal circumference. Over this was placed a dressing of carbolated cotton — this held in place by a wide flannel bandage.

The patient reacted nicely from the anæsthetic and shock, and for six hours appeared to be doing extremely well. Soon, however, I detected a failure in the volume of the radial pulse, which led to the suspicion that passive hæmorrhage was taking place from the myriad minute vessels which had been torn in breaking up the abdominal adhesions. Examination confirmed this suspicion, and, in spite of every effort to check this, the patient expired in four and one-half hours, from exhaustion. Notwithstanding the various aspirations, the tumor weighed 31.63 kilograms. A small sac at the posterior part of the tumor was found to contain nearly five kilograms of clear pus.

The chief regret is that the case was not seen at an earlier date, before disease had so far exhausted the strength of the patient that the tonic contractions of the minute vessels might have persisted in controlling the hæmorrhage.

*Case VI.* Was called Nov. 13th, 1878, to see Mrs. S. F., aged 58. Multipara, small and active. I found the patient had been suffering. for some two years, with severe epigastric neuralgia, pain in the back and nervousness. Examination revealed a fluctuating tumor extending from the cul-de-sac of Douglass to the ensiform cartilage. Wave motion was not distinct. The womb and bladder were drawn far up out of place; the margin of the os being two centimeters above the crest of the pubis. I aspirated from the vagina and drew off one kilogram of clear transparent fluid, the consistence of glycerine. With the needle still *in situ*, after having emptied the sac, attached the negative pole of an 18 cell zinc-carbon battery with the positive over the abdomen, I allowed the current to flow ten minutes.

November 17th. Circumference at the most prominent part of the tumor has decreased fifteen centimeters ; passed the continuous current, from vagina to umbilicus, with 18 cells. November 23d, I aspirated at the umbilicus and evacuated another sac containing, one kilogram of clear amber-colored fluid and passed the same current for ten minutes from aspirator needle to sacrum. November 30th, I aspirated in left superior inguinal region and obtained only a small amount of reddish serum, and passed the same current through the tumor. The abdominal dimensions diminished eight centimeters. December 4th, I aspirated in the right umbilical region and obtained two kilograms of clear serum. I sent a specimen of this fluid, and also that from case V., to Prof. Wm. H. Byford, of Chicago, who, without any data whatever, from microscopical examination, confirmed my diagnosis of ovarian cyst in case V. and of ovarian and ligamentous cysts in case VI. December 17th, I aspirated from the vagina and drew off one kilogram; the patient complained that the treatment following this aspiration gave more pain than usual. December 26th, I aspirated and drew off two kilograms of serum; the tumor has decreased four centimeters since last treatment. January 2d, I saw the patient, who was suffering from intense pain in the small of the back; urine scanty and high colored, bowels constipated for several days, some febrile symptoms and chills. I gave quinine to counteract any malarial or septic poison and morphia to relieve pain ; also ordered a laxative.

January 5th: The bowels have not moved, urine scanty; I introduced a catheter, which withdrew only a small quantity; I ordered a stronger cathartic and an injection in case the latter failed; pain less and fever not so marked; no chill since last visit. January 8th: No movement; some vomiting; injection was a complete failure; otherwise the patient is better; attached stomach tube to syringe and attempted to pass well up into the colon; found resisting obstructions ten or fifteen centimeters from rectum, beyond which neither tube nor fluid could be passed; hot fermentations were ordered over the bowels, in hopes of relaxing the parts. January 10th I found the patient but slightly rallied, she had had no movement; urine slightly better; on explaining the probable complication to the patient and family and advising an immediate operation, as the only possible chance of prolonging life, they at once consented; with the assistance of Dr. S. W. Gillespie, the only medical gentleman at hand, and the very able help of Mrs. Z. Hawley, I proceeded to operate under the carbolized spray. On opening the abdominal cavity in the usual manner, the displaced uterus and bladder presented upon the anterior aspect of the tumor; these organs were then pushed as much as possible out of the way and the dense adhesions of the tumor to the peritoneum were broken up with the hand. This having been carefully and expeditiously accomplished, the division of the broad ligament was commenced, and he who has attempted to remove the entire broad ligament in a cystic degeneration with its vessels enlarged and engorged, can well appreciate the task. With the dense adhesions in the cul-de-sac of Douglas, and the careful separation of the posterior membranes from the pelvic viscera, the time consumed in the removal was forty minutes. The right ovary, which formed a portion of the cystic mass, was next lifted up, its pedicle transfixed and tied each way. The cysts, fifteen in number, were then evacuated with the "Spencer Wells" trochar, the pedicle cut and secured, and the mass lifted out of the abdominal cavity. In doing this, the pressure ruptured a sac at the posterior aspect of the mass, and allowed a large quantity of decomposed pus to escape into the pelvic and abdominal cavities, bathing the freshly torn surfaces with this morbid agent. This was at once sponged out with warm carbolated water, and after ascertaining that there were no bleeding twigs, the walls were approximated with eight silver wire sutures, a strong strip of adhesive strap applied between each suture, a layer of carbolated cotton placed over the the incision and the whole secured by a wide flannel bandage.

The patient rallied well from the anæsthetic and operation, and, upon being told of the accident, replied she was glad the attempt had been made, and that it was an accident no care could have guarded against. After this, the patient conversed with her friends and left directions, messages, etc. About six hours from the time of the completion of the operation she began to fall into a lethargic condition, which continued to increase in spite of all efforts to stimulate, and ten hours from the operation death put an end to the scene, the patient falling into a profound coma. There was no hæmorrhage after the operation, and although, of necessity, there was considerable during that time, yet not enough to materially weaken the patient; every step being finished before the next was commenced.

Was the lethargic coma the result of the rapid absorption of pus by the peritoneal vessels or was it the result of exhaustion? The pulse was good until within two or three hours of death, and there were no signs of weakness; nothing except the heavy breathing, growing slower and slower until it ceased entirely. The reaction from the anæsthetic was most perfect.

Although the course pursued in the two cases last recorded was not crowned with success, yet the operations were just as imperative, and perhaps more so, than in the previous cases. For, in these two cases, operative procedure was the only chance of evading certain, speedy and painful death. While the chances were, of course, very much against recovery, yet we should avail ourselves of that chance whenever practicable.

While it is to be regretted that in case V the true pathology was not sooner understood, and, while in the condition in which the patient was found, death was the *probable* result of an operation, yet, if left to nature, the same result would have been sure and speedy.

In case VI, with the complication of an ovarian and ligamentous cyst, procrastination was one of the fatal factors. Although the necessary laceration of peritoneal tissue in the removal of the entire broad ligament caused by the large dense adhesions, was a grave factor, yet it was not necessarily fatal. This case had been diagnosticated during a period of two years as ovaritis, fibroma, sarcoma, retro-uterine hematocele, and even pregnancy. Amidst all these conflicting diagnoses, the wonder is that the true pathology was not stumbled upon. In this case the query arises, would it not have been better to have operated at once instead of attempting palliative measures? The weight of authority seems to be, in similar cases, to aspirate and only to operate as a last resort. In this case it is highly probable that the repeated aspirations favored the formation of the extensive and dense adhesions, by setting by circumscribed points of sub-acute peritonitis. Although there was no evidence of this, either upon the anterior or inferior posterior aspect of the tumor—the adhesions being universally dense and compact—yet the fear that this might have been instrumental in producing the result, prompts us to examine the case critically before dismissing it from our minds. The adhesions in the true pelvis were well marked when the case was first seen, and the commencement was referred by the patient to exposure and an uncomfortable position sustained during a journey of ten miles on an intensely cold day. Three vaginal aspirations were made during the treatment, and a careful examination gave no indication of their locality, on inspection after the operation; leading to the conclusion that their local results had disappeared.

